# TRAINERWALL: An Innovative, Cost-Effective Removable Anteroom for Pathogen Containment in Healthcare Settings

**DOI:** 10.3390/ijerph22040468

**Published:** 2025-03-21

**Authors:** Giorgio Ramorino, Anna Gobetti, Elena Roca, Giovanna Cornacchia

**Affiliations:** 1 Department of Industrial and Mechanical Engineering, University of Brescia, Via Branze 38, 25123 Brescia, Italy; giorgio.ramorino@unibs.it (G.R.); giovanna.cornacchia@unibs.it (G.C.); 2 Head and Neck Department, Neurosurgery, Istituto Ospedaliero Fondazione Poliambulanza, Via Bissolati 57, 25124 Brescia, Italy; rocaelena@gmail.com

**Keywords:** infection control, portable anteroom, pathogen containment, healthcare infrastructure, Personal Protective Equipment (PPE), donning and doffing, antimicrobial materials, sanitization mechanisms, resource-constrained settings

## Abstract

The COVID-19 pandemic exposed critical gaps in healthcare infrastructure, particularly the lack of effective isolation anterooms for managing infectious diseases. This study presents TrainerWall, a cost-effective, portable anteroom designed for rapid deployment in both high-resource and resource-limited settings. TrainerWall features a modular, self-supporting structure with antimicrobial materials, integrated sanitization, and adaptable dimensions, ensuring seamless integration into healthcare environments without disrupting operations. Its dual function as an infection control measure and a training platform makes it particularly valuable for non-specialized healthcare workers. Pre-clinical evaluations conducted in simulated hospital environments have demonstrated their ease of deployment, procedural efficiency, and positive impact on infection control practices, particularly in non-specialized departments. Designed for quick assembly, disassembly, and transport, TrainerWall enhances healthcare readiness during outbreaks, offering a scalable solution where permanent infrastructure is lacking. Future integration of augmented reality and interactive guidance could further improve training and compliance. This innovation provides an accessible, adaptable approach to infection control, particularly in underserved regions and emergency response scenarios.

## 1. Introduction

The COVID-19 pandemic has underscored the importance of effective infection control measures in healthcare settings worldwide, revealing critical gaps in the preparedness of healthcare facilities to manage highly infectious diseases [1,2]. One of the most significant deficiencies highlighted was the lack of appropriate isolation infrastructure, particularly the availability of anterooms that serve as transitional buffer zones to prevent the spread of pathogens between contaminated and clean areas. Anterooms play a pivotal role in infection control protocols by facilitating safe donning and doffing of Personal Protective Equipment (PPE) and by providing a space for hand hygiene, decontamination, and air filtration.

### 1.1. Current Challenges

Existing isolation solutions in healthcare environments primarily include permanent anterooms integrated into negative pressure isolation rooms, mobile isolation units, and temporary structures such as tents or modular pods [3]. While these systems are effective to some extent, they present several significant limitations that can reduce their utility, particularly during emergency scenarios such as pandemics.

- Permanent Anterooms: These are often constructed as part of specialized negative pressure isolation rooms. While effective in controlling the spread of airborne pathogens, they require substantial infrastructure investment, time, and specialized design, making them less feasible in rapidly evolving outbreak scenarios or in resource-limited settings. Moreover, their fixed nature limits the flexibility needed to quickly repurpose spaces to meet varying infection control demands. Studies have demonstrated that, although effective in well-prepared environments, isolation rooms with anterooms may fall short in less-resourced settings due to the costs and infrastructure required [4,5].
- Mobile Isolation Units: These units, such as negative pressure isolation pods and portable containment systems, offer some degree of flexibility and can be quickly deployed. However, they often lack adequate facilities for the safe handling of PPE and decontamination procedures. Additionally, they are usually expensive, require specialized training for setup and maintenance, and may not be readily available during sudden outbreaks. These logistical limitations hinder their adoption in resource-constrained environments or large-scale emergencies [6,7].
- Temporary Structures: Solutions like tents or makeshift barriers can be deployed quickly during health emergencies, but they generally lack the durability, proper ventilation, and structural integrity required for sustained use in healthcare settings. Although useful for rapid response, these temporary structures often fail to ensure strict adherence to infection control protocols, such as proper air filtration and sanitation measures, potentially compromising the safety of healthcare staff and patients. Studies have highlighted that while these solutions provide immediate relief, they are unable to maintain long-term safety standards [8,9].

Specifically, the study highlights three major deficiencies in current infection control infrastructure:

- Lack of adaptable and rapidly deployable anterooms—Many healthcare facilities, particularly in non-specialized departments and resource-limited settings, lack dedicated isolation buffer zones, increasing the risk of cross-contamination.
- Limited cost-effective solutions—Permanent anterooms and mobile isolation units often require significant investment and infrastructure, making them impractical for emergency response or low-resource environments.
- Insufficient standardized PPE donning/doffing areas—The absence of dedicated decontamination spaces contributes to procedural errors and higher infection risks among healthcare workers.

Given these limitations, there is a clear need for more versatile, cost-effective, and easy-to-deploy solutions that can be rapidly installed in both high-resource and resource-limited settings. These solutions should ensure strict adherence to infection control protocols while offering better protection for healthcare workers and patients.

Additionally, the effectiveness of existing isolation systems is closely tied to the proper use of PPE within well-structured environments. Personal protective equipment, such as gowns, gloves, goggles, and boots, is critical for safeguarding healthcare workers (HCWs) from infectious agents. However, the effectiveness of PPE largely depends on its correct usage and removal, which must take place within a controlled environment [10]. Such an environment serves as essential “filter zones”, designed to separate “dirty” (contaminated) areas from “clean” areas, thereby preventing cross-contamination. Fatigue, insufficient training, and the absence of properly designated spaces for donning and doffing PPE significantly contribute to procedural errors. These factors were especially pronounced during the COVID-19 pandemic, exacerbating the rates of healthcare-associated infections (HAIs) among medical personnel.

Ventilation systems play a fundamental role in infection prevention and control within healthcare facilities, particularly in isolation areas. Current World Health Organization’s guidelines establish specific standards for airborne infection isolation rooms, requiring a minimum ventilation rate of 160 L/s/patient and at least 12 air changes per hour in new construction, while accepting six air changes per hour (ACH) in existing facilities [11]. In a comprehensive study of 45 hospitals during the COVID-19 pandemic, Tang et al. [12] demonstrated that negative pressure rooms operating at >12 ACH effectively reduced airborne viral particles by 99% within 30 min. These findings underscore the critical importance of proper ventilation design and maintenance in both permanent and temporary isolation facilities for effective infection control.

During the first wave of the pandemic, as of 20 April 2020, a total of 17,997 healthcare workers were infected with the SARS-CoV-2 virus in Italy alone [13]. Although the data do not specify the professions involved, it is probable that even in hospital environments, there was transmission of the virus due to surface and airborne contagion in a filter environment that was not perfectly suited to the type of virus. Infected patients may have inadvertently transmitted the virus to healthcare workers, who in turn may have spread it to colleagues and into the wider communities. Surface contact with contaminated areas remains a possible transmission route, though the primary mode of transmission for coronaviruses is airborne, as confirmed by recent studies. While surface contamination can contribute to the spread, several factors, such as temperature, material type, and duration of exposure, influence its risk [14].

Moreover, departments that are not specialized in infectious disease treatment often struggle to establish and maintain effective filter zones that separate contaminated from non-contaminated areas. These areas are critical for auxiliary patient care activities and the safe management of PPE. The situation was further complicated as healthcare professionals from diverse specialties were often thrust into high-pressure situations, working with highly contagious patients in an exceptional pandemic or emergency context. This scenario increased their likelihood of exposure to pathogens, particularly when compared to colleagues in infectious disease units, who are more experienced and better trained in managing biological risks.

The absence of specialized facilities, standardized disinfection protocols specific to COVID-19, and proper PPE-offing procedures have significantly contributed to the transmission of infections within healthcare settings. Although COVID-19 cases have declined globally, viral epidemics continue to represent a substantial threat in various regions, particularly developing countries. In these areas, effective containment of infectious pathogens remains a critical challenge. Many healthcare infrastructures are still under-resourced, lacking the necessary tools and facilities to adequately manage and control the spread of highly infectious viruses such as COVID-19 and Ebola. As such, the development of innovations aimed at enhancing infection control practices remains as urgent and relevant today as it was during the height of the COVID-19 pandemic.

### 1.2. Proposed Solution: TrainerWall

This study presents an innovative solution to these ongoing challenges: the TrainerWall, a portable filtration chamber designed to function as a removable anteroom. TrainerWall provides a rapid, adaptable, and cost-effective means of establishing controlled, contamination-free environments within healthcare settings.

The TrainerWall is designed for easy assembly and disassembly, enabling it to be deployed across various hospital settings without impeding the movement of stretchers or other essential equipment. The system is reusable and can be sanitized for ongoing use or repurposed for field training in infection control practices. Its design is intended to minimize operational errors, incorporating features and optional technologies that guide users in adhering to correct procedures based on established health protocols. This study aims to demonstrate how TrainerWall could serve as an affordable and practical solution for reducing pathogen transmission within healthcare environments. Additionally, it highlights its potential to enhance biosafety during both routine and emergency operations, particularly in regions with limited healthcare resources and a high risk of viral outbreaks. TrainerWall is a system that has not yet undergone clinical trials due to the necessary regulatory approvals, but it has been pre-clinically evaluated in simulated hospital environments. In these environments, healthcare professionals assessed its usability, efficiency, and practical benefits. Their qualitative feedback indicates that TrainerWall could significantly enhance infection prevention measures, improve procedural adherence, and support healthcare facility readiness, particularly in non-specialized departments where existing isolation solutions are limited.

### 1.3. Objectives

The primary objective of this study is to illustrate the TrainerWall system as a simple, economical, and dual-function solution for infection control in healthcare settings. The system aims to:

- Provide a rapid and adaptable isolation solution: Demonstrate the ease of assembly, disassembly, and transport of the TrainerWall system, enabling quick deployment in various healthcare settings, including resource-limited environments.
- Enhance infection control practices: Showcase the system’s ability to facilitate safe donning and doffing of PPE through integrated sanitization mechanisms and antimicrobial materials, thereby reducing the risk of pathogen transmission (Appendix A).
- Serve as a training platform: Highlight the dual functionality of TrainerWall as both an active infection control measure and a training tool for healthcare workers, particularly in underserved areas, to improve compliance with infection control protocols.
- Promote operational efficiency: Illustrate how the system’s design minimizes operational errors and streamlines infection control procedures, contributing to a safer working environment for healthcare personnel.

By achieving these objectives, the TrainerWall system aims to address the critical challenges in infection control and provide a flexible, accessible solution to enhance healthcare facility readiness for managing infectious disease outbreaks worldwide.

## 2. Materials and Methods

### 2.1. Materials

#### 2.1.1. Structural Design and Components

**Figure 1:**
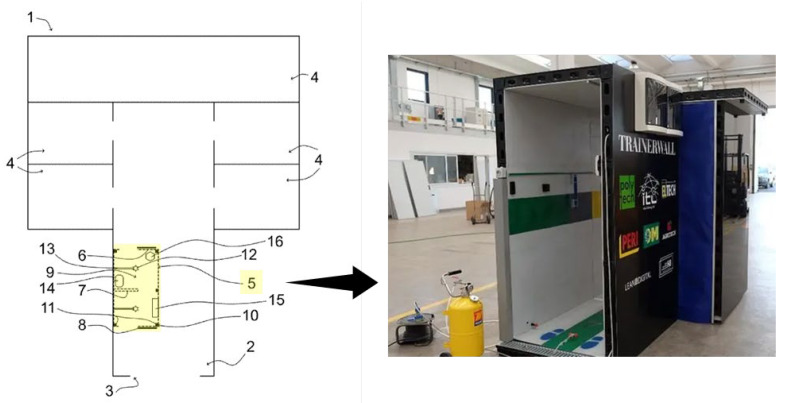
TrainerWall system: (1) modular anteroom structure; (2) central corridor; (3) entrance; (4) infected patients rooms; (5) modular structure (TrainerWall); (6, 7) multiple interconnectable walls; (8) optional door; (9) anteroom space; (10) frame system; (11) aluminum profiles; (12) nozzles to spray a liquid sanitizing agent; (13) equipment sanitization system; (14) cabinet and sink; (15) PPE dispenser system; (16) container for the safe disposal of potentially contaminated PPE [18,19].

The TrainerWall system, illustrated in Figure 1, offers an innovative solution for establishing temporary isolation anterooms within healthcare environments. Designed for deployment in existing hospital wards (1) that lack dedicated buffer zones, the system is particularly useful in situations where patients with highly infectious diseases are housed in standard rooms (4).

At the core of the system is a modular structure (5) composed of multiple interconnectable walls (6, 7). These walls can be easily assembled to create a functional anteroom space (9), acting as a critical buffer zone between contaminated areas and clean hospital corridors (2). The flexibility of the design allows for additional partitioning within the anteroom, using dividing walls (e.g., wall 7), to establish distinct zones for different stages of the PPE donning and doffing process.

#### 2.1.2. Structural Integrity and Adaptability

The TrainerWall system is engineered for both structural integrity and flexibility. The walls (6, 7) are designed to be self-supporting, standing vertically on the hospital corridor floor without the need for external reinforcement. For enhanced stability and adaptability, the system includes an aluminum frame system (10) constructed from lightweight yet robust aluminum profiles (11). This frame allows for mechanical anchoring of the walls, significantly increasing the structural integrity of the entire setup.

A key feature of the TrainerWall is its dimensional adaptability. The aluminum frame profiles (11) can be supplied in various lengths or designed with a telescopic mechanism, allowing for adjustments in length, width, and potentially height. This flexibility ensures that the system can be easily tailored to corridors and rooms of different dimensions, while still maintaining critical functionality. Importantly, the structure is designed not to obstruct the passage of stretchers or hospital beds, a vital consideration in busy healthcare settings.

#### 2.1.3. Materials and Sanitation

The walls (6, 7) and optional doors (8) of the TrainerWall system are constructed from aseptic materials that can be readily sanitized. The preferred choice is polyethylene that has been treated in a hydrogen peroxide bath. Polyethylene is selected for its antimicrobial properties and its ease of cleaning, which are beneficial in a variety of settings. This material is crucial in maintaining a sterile environment within the anteroom, reducing the risk of pathogen transmission during both routine and emergency use. The scientific literature and infection control guidelines emphasize that smooth, non-porous surfaces treated with antimicrobial agents can significantly reduce microbial colonization and facilitate routine decontamination [15]. In TrainerWall, the use of high-quality injection-molded plastic panels treated with hydrogen peroxide is intended to create surfaces that resist microbial adherence and support efficient cleaning protocols.

Moreover, the selection of aluminum for the system’s adjustable frame is based on its lightweight properties, excellent durability, and resistance to corrosion. This makes aluminum alloy an ideal material for environments where strong disinfectants are frequently used, such as healthcare settings. These characteristics are especially valuable for a modular system that is assembled and disassembled frequently, as highlighted in studies on materials used in healthcare environments. Additionally, aluminum components are also easier to clean and maintain, contributing to the overall reliability and longevity of the device.

To further enhance infection control, the TrainerWall system incorporates an integrated sanitization mechanism. This system includes strategically placed nozzles (12) within the anteroom (9), designed to disperse a liquid sanitizing agent—typically hydrogen peroxide—across the surfaces of the walls (6, 7) and doors (8). The sanitizing solution is supplied via tubing (13) connected to an external tank and pump system, enabling automated, uniform disinfection of the entire anteroom after each use. This feature ensures a high level of sanitation is maintained with minimal manual intervention, making the system both effective and efficient in controlling contamination.

According to Istituto Superiore di Sanità (ISS) [16] and World Health Organization (WHO) [17] guidelines for infection control, standard precautions include the following:

- Hand Hygiene: TrainerWall facilitates effective hand hygiene by incorporating integrated sink and sanitizer dispensers within the isolation space, ensuring that healthcare workers can perform handwashing or hand sanitization immediately before and after entering the space.
- Use of Personal Protective Equipment (PPE): TrainerWall is designed with a dedicated area for the safe donning and doffing of PPE. This controlled environment minimizes cross-contamination by ensuring that healthcare workers adhere to PPE protocols consistently.
- Respiratory Etiquette: Although respiratory etiquette—such as covering the mouth and nose during coughs or sneezes—is not directly managed by TrainerWall, the system’s design minimizes interpersonal contact and supports a well-organized space, indirectly reducing the risk of respiratory droplet transmission.
- Sharps Safety: TrainerWall includes designated zones and secure disposal systems for sharps, reducing the risk of accidental injuries and ensuring that contaminated materials are handled and discarded safely.
- Safe Injection Practices: By providing clearly defined, contamination-free zones, TrainerWall supports safe injection practices. The controlled environment aids healthcare workers in performing injections with minimal risk of exposure or cross-contamination.
- Instrument Sterilization: While instrument sterilization is not a function of TrainerWall, it focuses on creating a clean, contamination-free environment that complements existing sterilization protocols used for medical devices.
- Disinfection and Cleaning of Environmental Surfaces: TrainerWall features an integrated automated sanitization mechanism that efficiently disinfects all surfaces within the anteroom after each use, ensuring that the area remains free of pathogens and compliant with rigorous cleaning standards.

These design features demonstrate TrainerWall’s compliance with standard infection control guidelines, ensuring that it not only meets but enhances critical safety measures in healthcare settings.

#### 2.1.4. Functional Design for Healthcare Procedures

The anteroom (9) is carefully designed to streamline and enhance the hygiene protocols surrounding the donning and doffing of PPE for healthcare personnel. Its layout and components are optimized to ensure safety, efficiency, and comfort. Key components include:

- Hand Hygiene Station: A cabinet and sink (14) equipped with electronic, touchless faucets to promote proper hand hygiene and minimize cross-contamination.
- Seating Area: A designed seating area, such as a bench or chair, to provide staff with comfort and stability during the donning process.
- PPE Dispenser System: A convenient dispenser system (15) that offers easy access to essential PPE items, including disposable shoe covers, gloves, gowns, FFP2/FFP3 masks, and protective eyewear.
- PPE Disposal: A specialized container (16) for the safe disposal of potentially contaminated PPE, helping to prevent environmental contamination and ensure proper waste management.
- Reusable PPE Sanitization: An optional sanitization system designed to disinfect reusable PPE items, further supporting the sustainability and safety of infection control measures.

This functional design not only supports effective infection control but also minimizes the risk of procedural errors, contributing to a safer working environment for healthcare personnel.

#### 2.1.5. Additional Features

To further enhance the functionality and safety of the TrainerWall system, various optional components can be integrated. These features are designed to improve ease of use, monitoring, communication, and procedural efficiency:

- Handles: Strategically placed handles to facilitate the movement of personnel and equipment within the anteroom, ensuring ease of navigation.
- Cameras: Integrated cameras for remote monitoring and training purposes, enabling real-time observation of healthcare procedures and improving compliance with infection control protocols.
- Speakers: Audio systems for delivering instructions, and facilitating clear communication between personnel inside and outside the anteroom.
- Lighting Sources: Adequate and strategically placed lighting to ensure optimal visibility during critical procedures, enhancing safety and precision.
- Information Panels and Whiteboards: Display panels and whiteboards for communicating essential information, such as current protocols, patient statuses, or PPE guidelines.
- Wiring and Supply Lines: Necessary electrical and data wiring to support the operation of cameras, speakers, and lighting, as well as the integration of additional technologies as needed.

These additional features are designed to make the TrainerWall system more versatile and user-friendly, providing healthcare workers with the necessary tools to maintain high standards of safety and efficiency, especially during critical or high-risk operations.

### 2.2. Methods

#### 2.2.1. Assembly

TrainerWall system is designed to be efficient and can typically be completed by two trained personnel. Below is a detailed, step-by-step guide for the assembly process:

1. Site Preparation:
  - Identify an appropriate location within the healthcare facility, typically a corridor (2) or near the entrance of a patient care area (1).
  - Ensure the site provides sufficient space for installation without obstructing critical pathways.
2. Frame Assembly:
  - If the adjustable frame (10) is used, assemble the frame by connecting the metal profiles (11). The profiles may be telescopic or come in various lengths to fit the installation site’s dimensions.
  - Ensure the frame is level and properly adjusted to the size of the available space.
3. Wall Panel Installation:
  - Attach the coupling wall panels (6, 7) to the frame, or position them as self-supporting units if the frame is not used.
  - Align the panels carefully to define the anteroom space (9) and create a secure barrier between the contaminated and clean areas.
4. Ceiling Installation (Optional):
  - If a ceiling is part of the design, install the upper panels to fully enclose the anteroom (9), providing additional containment and environmental control.
5. Door Installation (Optional):
  - Install sliding doors (8) at designated entry and exit points. Automated doors are preferable to reduce contact and enhance infection control.
6. Anchoring:
  - Secure the structure to the floor and adjacent walls using appropriate fasteners, such as anchor bolts, ensuring the TrainerWall is stable and resistant to external pressures.
7. Equipment Integration:
  - Install essential fixtures and equipment within the anteroom, including:
    ○ Sink (14) for hand hygiene.
    ○ Dispensers (15) for sanitization products and PPE.
    ○ Waste collection system (16) for the safe disposal of contaminated materials.
    ○ Additional optional features, such as handles, cameras, speakers, mirrors, light sources, information panels, and whiteboards, can be added as needed.

#### 2.2.2. Sanitation System Setup

- Connect the automated sanitation system, including the nozzles (12) and tubing (13), to external reservoirs and pump systems. This setup allows for efficient delivery of disinfectants, such as hydrogen peroxide, to sanitize the anteroom space after each use.

1. Electrical and Plumbing Connections:
  - Complete any necessary electrical and plumbing connections for the sink, lighting, and other integrated equipment.
  - Ensure that the connections meet the required healthcare standards for safety and hygiene.
2. Final Inspection:
  - Conduct a thorough inspection of the assembled TrainerWall system.
  - Verify the structural integrity, check the functionality of all installed components, and ensure that the corridor or patient care area can still accommodate the passage of stretchers or hospital beds without obstruction.

By following these detailed assembly steps, the TrainerWall system can be effectively deployed in various healthcare settings, offering flexible, robust, and rapidly deployable solutions for infection control, biosafety, and pathogen containment.

## 3. Operation

### 3.1. Recommended Standard Procedure for Donning and Doffing

The recommended protocol for healthcare personnel when using PPE aims to ensure thorough protection and minimize contamination risk. This procedure involves several critical steps for both donning and doffing PPE.

Donning Procedure:

Before donning PPE, healthcare workers should remove all personal items, including jewelry, and perform hand hygiene using soap and water or an alcohol-based sanitizer. Protective shoe covers are then worn to prevent surface contamination. It is essential to inspect all PPE components, such as gloves, gowns, masks, and goggles, for any defects and replace any damaged items. The first pair of gloves is applied, ensuring proper fit and coverage, followed by wearing a disposable gown over the uniform. Facial filtering protection, such as an FFP2/FFP3 mask, and protective goggles, are then donned. Finally, a second pair of gloves is applied over the first pair to provide an additional layer of protection.

Doffing Procedure:

During the doffing process, it is crucial to minimize contact with potentially contaminated PPE, avoiding touching the face, mucous membranes, or skin. Disposable PPE, including gowns and gloves, should be placed in designated biohazard containers, while reusable items like goggles must be sanitized promptly. The sequence of removal involves first removing and discarding the gown by rolling it inward to contain contamination. Next, the first pair of gloves is removed and disposed of, followed by carefully removing and sanitizing the goggles. The FFP2/FFP3 mask is then removed by handling it from the back and disposing of it. Finally, shoe covers and the second pair of gloves are removed, followed by performing hand hygiene with soap and water or an alcohol-based sanitizer.

Despite the detailed and structured approach to PPE use, several challenges and criticisms have emerged. The absence of dedicated anterooms or filter zones in many healthcare facilities has made effective implementation of these procedures challenging. Hospitals have infectious disease departments that are specifically designed to contain the contagion; however, in the remaining departments, there are no such measures since infected patients are usually centralized in the infectious disease departments. In the event of a pandemic, such as during the COVID pandemic, hospital facilities have therefore had to deal with a situation of high contagion that has involved the entire hospital, including departments that are not suitable for the control of highly contagious infectious diseases. This shortcoming is compounded by the fact that severe pandemics like COVID-19 are relatively rare, and therefore, healthcare infrastructures were unprepared for such scenarios. The complexity and time-consuming nature of the donning and doffing procedures can lead to fatigue and potential errors, particularly during extended shifts or emergency situations. This increases the risk of contamination if protocols are not meticulously followed. Furthermore, the procedures assume the availability of all necessary PPE components, which may be unrealistic in resource-limited settings or during supply shortages.

Additionally, the debate surrounding the effectiveness of double gloving suggests that while it provides an extra layer of protection, it may also reduce dexterity without significantly improving overall safety [20]. The reuse and decontamination of certain PPE items, though necessary in some contexts, raise concerns about the degradation of their protective properties over time. Lastly, the psychological stress associated with rigorous PPE protocols, especially the fear of self-contamination during doffing, can impact healthcare workers’ mental well-being and potentially lead to avoidance behaviors.

These criticisms highlight the need for ongoing refinement of PPE protocols and the development of more user-friendly, efficient, and universally applicable infection control solutions. Systems like TrainerWall aim to address these issues by offering a practical, adaptable, and efficient approach to infection management, capable of enhancing biosafety in diverse healthcare settings.

These challenges underscore the need for innovative solutions to streamline PPE procedures and enhance infection control. Systems like TrainerWall aim to address these issues by offering a practical, adaptable, and efficient approach to infection management in diverse healthcare settings.

### 3.2. Procedure Using TrainerWall

The utilization of TrainerWall introduces a streamlined approach to the donning and doffing procedures, significantly enhancing both efficiency and safety compared to traditional methods.

TrainerWall integrates interactive guidance systems and augmented reality (AR) technologies to provide real-time, step-by-step instructions to healthcare personnel.

Before entering the patient care area, healthcare personnel interact with TrainerWall to prepare for PPE use. The system prompts them to remove all jewelry and personal items, perform meticulous hand hygiene with either soap and water or an alcohol-based solution, and don protective shoe covers. TrainerWall then verifies the integrity of all PPE components and prompts personnel to don the first pair of gloves. Unlike traditional methods, where personnel manually check and put on each item, TrainerWall ensures each step is accurately followed through visual and auditory cues. This reduces the risk of errors and enhances compliance with safety protocols. To ensure compliance with infection prevention protocols, the TrainerWall system is designed to accommodate one person per session. This measure aligns with minimum distancing requirements, reducing the risk of cross-contamination during the donning and doffing of PPE. By limiting access to a single healthcare worker at a time, the system maintains a controlled environment, optimizing the effectiveness of decontamination procedures and ensuring adherence to biosafety standards.

During the doffing process, TrainerWall continues to guide personnel through the sequence of removing and disposing of disposable PPE and decontaminating reusable equipment. AR overlays highlight critical steps, such as the correct sequence for removing the gown and gloves to prevent cross-contamination. Immediate feedback is provided on their actions, reinforcing proper technique and ensuring thorough decontamination. By integrating TrainerWall into the workflow, healthcare facilities benefit from standardized procedures that enhance infection control measures and minimize the risk of healthcare-associated infections. The interactive nature of TrainerWall not only improves procedural adherence but also accelerates the training process for new staff, making it a valuable tool in modern healthcare settings.

Compared to traditional methods that rely on manual checks and written protocols, TrainerWall offers a dynamic solution that adapts to evolving healthcare needs. It enhances the overall safety and efficiency of PPE management through its interactive and adaptive features.

#### Sanitization Procedure

The sanitization procedure is a critical component of the TrainerWall system, ensuring that the anteroom remains a sterile and safe environment for healthcare personnel. The integrated sanitization mechanism is designed to automate the disinfection process, minimizing manual intervention and reducing the risk of human error. Below is a detailed description of the sanitization procedure:

1. Preparation:
  - Ensure that the external reservoir containing the sanitizing agent (typically hydrogen peroxide) is adequately filled.
  - Connect the tubing (13) from the reservoir to the nozzles (12) within the anteroom (9).
  - Verify that the pump system is functional and ready to dispense the sanitizing agent.
2. Activation:
  - Initiate the sanitization cycle using the control panel or automated system integrated into the TrainerWall.
  - The pump system will activate, drawing the sanitizing agent from the external reservoir and distributing it through the tubing (13) to the nozzles (12).
3. Dispersion:
  - The nozzles (12) will spray the sanitizing agent evenly across the surfaces of the walls (6, 7) and doors (8) within the anteroom (9).
  - Ensure that the dispersion covers all critical surfaces, including areas frequently touched by healthcare personnel.
4. Contact Time:
  - Allow the sanitizing agent to remain on the surfaces for the recommended contact time to ensure effective disinfection.
  - The contact time may vary depending on the specific sanitizing agent used and the level of contamination.
5. Rinsing (if necessary):
  - If the sanitizing agent requires rinsing, use the integrated rinsing system to remove any residual sanitizer from the surfaces.
  - Ensure that the rinsing process is thorough to prevent any potential skin irritation or material degradation.
6. Drying:
  - Allow the surfaces to air dry completely before the anteroom is used again.
  - Proper drying is essential to maintain the effectiveness of the sanitizing agent and prevent the growth of pathogens.
7. Final Inspection:
  - Conduct a visual inspection of the anteroom to ensure that all surfaces have been adequately sanitized.
  - Check for any residual sanitizing agent or moisture that may require additional attention.

By following this detailed sanitization procedure, the TrainerWall system ensures a high level of hygiene and safety within the anteroom, contributing to the overall effectiveness of infection control measures in healthcare settings.

**Figure 2:**
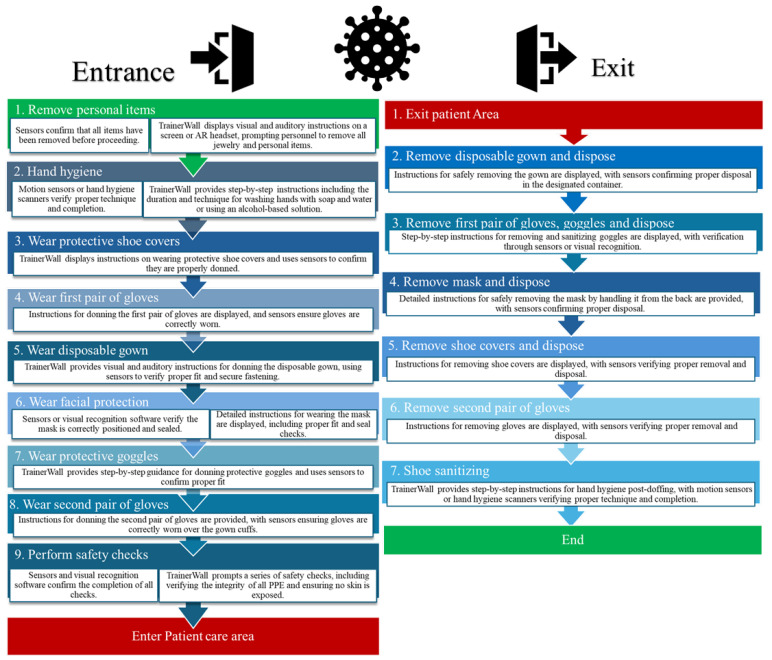
Flow chart of the TrainerWall guided procedure to access and exit contaminated areas.

**Figure 3:**
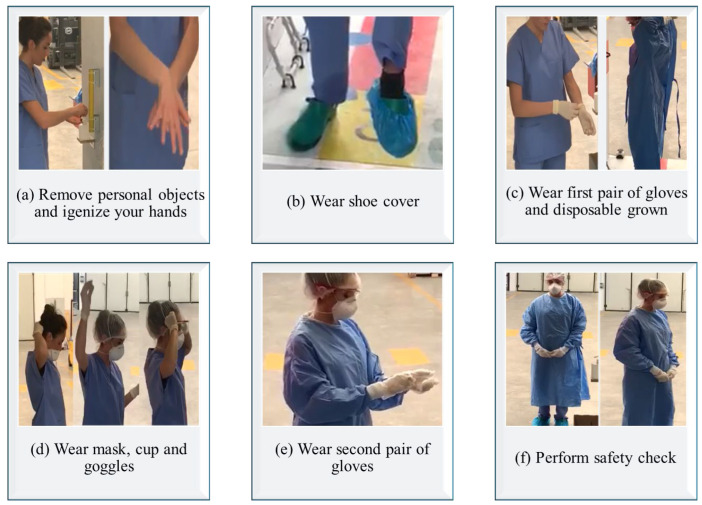
Key sequences of the donning procedure from the experimentation conducted on the TrainerWall prototype.

**Figure 4:**
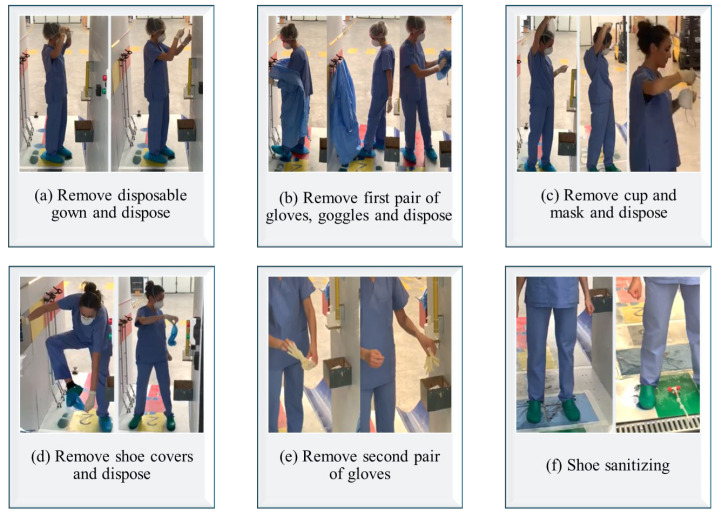
Key sequences of the doffing procedure from the experimentation conducted on the TrainerWall prototype.

Figure 2 illustrates the flow chart of TrainerWall guided procedure for accessing and exiting contaminated areas. Figure 3 and Figure 4 show key sequences of donning and doffing procedures, respectively, from the experimentation conducted on the TrainerWall prototype.

By providing a structured and technologically advanced approach, TrainerWall addresses many of the limitations inherent in traditional PPE management methods, ultimately contributing to better infection control and enhanced safety in healthcare environments.

## 4. Discussion

The TrainerWall system presents a novel and potentially transformative approach to infection control in healthcare environments, addressing several critical limitations inherent in traditional isolation methods [21]. Although quantitative preperformance data are not yet available, the system’s design principles, functionality, and practical applications align with current best practices in pathogen containment and personnel safety, suggesting its efficacy in real-world settings. Based on its design principles, functionality, and practical applications, the system aligns with best practices in pathogen containment and personnel safety, suggesting it can perform effectively in real-world scenarios [22].

One of the most compelling aspects of the TrainerWall system is its modular and highly adaptable structure. In environments that lack permanent buffer zones, such as older hospitals or makeshift facilities, TrainerWall can be rapidly assembled to create effective contamination barriers. This capability is particularly crucial during pandemics or localized outbreaks when hospitals experience surges in patients with infectious diseases. Unlike conventional construction-based buffer zones, which are time-consuming and costly to install, TrainerWall provides an easily deployable alternative, ensuring that healthcare facilities can quickly establish critical isolation spaces without disrupting the flow of patients and staff. Moreover, the system’s adjustable dimensions make it highly versatile, allowing it to fit into different hospital spaces without compromising access to essential equipment such as stretchers or medical carts. This adaptability is vital in maintaining operational efficiency, particularly in high-traffic areas such as corridors, where space constraints often pose challenges. By preserving mobility and access, TrainerWall ensures that patient care activities can continue without compromising the safety and sterility of isolation zones.

TrainerWall’s use of polyethylene treated with hydrogen peroxide for the wall panels is a strategic choice aimed at maximizing asepsis. Polyethylene’s inherent resistance to microbial colonization, when enhanced by hydrogen peroxide treatment, provides a dual defense against contamination. The material’s smooth, non-porous surface facilitates easy cleaning, and its durability ensures it can withstand repeated disinfection cycles without degradation [15]. This feature addresses a common limitation in temporary isolation systems, where material wear and difficulty in maintaining sterility can undermine infection control efforts. The integrated automated sanitation system further strengthens and enhances the system’s ability to maintain a sterile environment. Nozzles strategically placed within the anteroom spray disinfectant, such as hydrogen peroxide, ensure a thorough and consistent disinfection process after each use. This automated process not only minimizes human error but also reduces the time healthcare workers spend on manual cleaning, which is both labor-intensive and prone to variability in efficacy. Automation also ensures a higher level of compliance with infection control protocols, particularly in high-pressure settings where time and accuracy are critical [23].

The TrainerWall system is designed to address one of the most significant risk points for healthcare workers: the donning and doffing of personal protective equipment. Studies have demonstrated that improper doffing is a contributor to self-contamination, which increases the risk of healthcare-associated infections [24]. TrainerWall’s design incorporates interactive guidance systems and, potentially, augmented reality technologies to ensure that each step of the donning and doffing process is followed meticulously. This feature reduces procedural errors by providing real-time feedback and visual cues, thereby enhancing compliance with safety protocols. Additionally, the inclusion of ergonomic elements such as seating areas, touchless sinks for hand hygiene, and strategically placed PPE dispensers streamlines the process and reduces physical strain on healthcare workers. These design enhancements are particularly valuable during extended shifts or emergency situations, where fatigue can lead to lapses in procedure. By facilitating an intuitive and user-friendly experience, TrainerWall promotes not only safety but also the well-being of healthcare personnel.

TrainerWall’s dual functionality as both an infection control barrier and a training platform adds to its overall value in healthcare settings. In addition to its role in preventing pathogen spread, TrainerWall serves as a controlled environment where healthcare workers can practice and refine their donning and doffing techniques. This capability is particularly important in regions where healthcare systems may be under-resourced or where training opportunities are limited. The ability to conduct training in a realistic, simulated environment prepares healthcare personnel for high-risk situations, ensuring they can perform critical procedures with confidence and precision. Furthermore, the potential integration of augmented reality technology into the training process represents a significant innovation. Augmented reality allows for immersive, hands-on learning experiences, enabling healthcare workers to visualize and rehearse complex procedures before encountering real-world scenarios. This kind of interactive training has been shown to enhance knowledge retention and reduce errors, making it a valuable tool in building a more resilient and prepared healthcare workforce. While the theoretical advantages of TrainerWall are clear, it is crucial to recognize that real-world effectiveness will depend on several factors, including the speed of assembly, user adoption, and measurable outcomes in pathogen transmission reduction. One potential limitation is the need for personnel to be trained in both the assembly and operation of the system, which may require initial time investments that could delay deployment during emergencies. However, the simplicity of the design, coupled with the step-by-step assembly guide, suggests that these hurdles can be easily overcome with proper planning and preparation. Further empirical research is needed to fully assess TrainerWall’s impact on infection rates. Studies that track infection rates among healthcare workers before and after the implementation of TrainerWall could provide valuable insights into its efficacy. Additionally, feedback from healthcare personnel who have used the system in practice would help refine its design and functionality, ensuring it meets the practical needs of frontline workers.

TrainerWall presents a cost-effective alternative to traditional isolation solutions, balancing affordability with functionality. With an estimated cost of approximately EUR 15,000, TrainerWall is significantly more economical than mobile isolation units, which can cost around EUR 47,000 per unit [25], and offers greater flexibility than permanent anterooms, which range between EUR 4800 and EUR 19,000 per room, depending on size and design complexity [5]. While temporary structures such as isolation tents are initially cheaper (EUR 1400–EUR 2400 per unit), they often lack the durability, ventilation, and structural integrity needed for sustained use in healthcare environments. TrainerWall, by contrast, is a modular, reusable, and easily deployable system, featuring an integrated sanitization mechanism that enhances infection control with automated disinfection. Additionally, its dual functionality as both an isolation barrier and a training platform makes it particularly valuable in resource-limited settings, where both containment measures and staff training opportunities are often scarce.

## 5. Future Research Directions

To further validate and enhance the TrainerWall system, specific future research directions include:

Regulatory Approvals and Clinical Validation: Since TrainerWall is a patented medical device, transitioning from a prototype to clinical implementation requires specific regulatory steps. The next phase involves obtaining Ethics Committee approval for clinical trials and securing authorization from the Ministry of Health as a non-CE-marked medical device. Once these approvals are in place, controlled clinical studies will be conducted to collect quantitative data on its impact on infection control, usability, and effectiveness.

Field Trials: Conducting field trials in various healthcare settings to assess the system’s real-world performance. These trials will focus on measuring its effectiveness in reducing pathogen transmission rates, improving infection control practices, and optimizing its usability in different medical environments, particularly in non-specialized departments that lack dedicated isolation infrastructure.

Modular Design Improvements: Exploring potential improvements in the modular design to enhance flexibility and adaptability. Research will focus on lightweight materials, improving ease of assembly and disassembly, and incorporating additional customization options to fit diverse healthcare environments more efficiently.

Cost Reduction Strategies: Investigating cost-reduction strategies to increase accessibility, particularly in resource-limited settings. This could involve alternative materials, optimized production processes, and leveraging economies of scale to ensure affordability without compromising quality.

Technological Integration: While TrainerWall is designed as a standalone solution for infection control and healthcare training, future iterations may incorporate augmented reality (AR) and interactive guidance systems to further enhance user experience and compliance with infection control protocols. These technologies could provide real-time visual instructions, reinforcing best practices during donning and doffing of PPE. However, recognizing the limitations of resource-constrained settings, AR integration remains an optional feature, ensuring that TrainerWall remains accessible and adaptable to different healthcare environments without requiring additional technological investments.

User Feedback and Training: Collecting feedback from healthcare personnel who have used TrainerWall in simulated and real-world settings. This qualitative input will inform improvements in user experience, ergonomics, and training effectiveness, ensuring that the system meets the practical needs of frontline workers.

Longitudinal Studies: Conducting longitudinal studies to track infection rates among healthcare workers before and after the implementation of TrainerWall. These studies will provide valuable data on the system’s efficacy over time and highlight areas for further refinement.

TrainerWall’s potential impact extends beyond individual healthcare facilities to address broader global health challenges. In resource-limited settings, it offers a practical and cost-effective solution for facilities lacking dedicated isolation infrastructure, potentially reducing healthcare-associated infections through its rapidly deployable anteroom system. The system’s dual functionality as both an infection control measure and training platform is particularly valuable in non-specialized departments and underserved regions, where it can improve compliance with PPE protocols and enhance safety procedures. Its modular and portable design ensures scalability across diverse healthcare environments, making it an effective tool for managing infectious disease outbreaks globally. Future iterations could incorporate advanced technologies such as augmented reality to provide real-time guidance, further enhancing training effectiveness and procedural adherence. These features position TrainerWall as a potentially transformative tool for global health efforts, particularly in regions with limited healthcare infrastructure.

By pursuing these research directions, TrainerWall can be further refined and validated, contributing significantly to global infection control efforts. This structured approach ensures that the system progresses from a validated prototype to a fully tested clinical solution, bridging the gap between concept, regulatory approval, and large-scale adoption in healthcare settings worldwide.

## 6. Conclusions

The TrainerWall system offers a promising solution to the pressing challenges of infection control in healthcare settings, addressing both immediate pandemic-related demands and long-term infectious disease management. Its modular and portable design provides an adaptable isolation solution for healthcare facilities that lack built-in anterooms, especially in resource-limited environments. The system’s rapid deployment, use of antimicrobial materials, and integrated sanitization features enhance its effectiveness as an infection barrier, while its dual functionality as a training platform further strengthens healthcare preparedness. TrainerWall’s versatility makes it particularly valuable for managing a wide range of infectious diseases, such as Ebola, tuberculosis, and cholera, which are prevalent in many developing countries. Its ease of assembly and portability enable healthcare workers, even those with limited specialized training, to quickly establish and maintain isolation zones during outbreaks, a flexibility that is crucial for remote or underserved areas where permanent infrastructure is inadequate or lacking. Furthermore, the system’s user-friendly design, combined with the potential integration of advanced technologies like augmented reality, offers a forward-thinking approach to enhancing compliance with infection control protocols. Key findings indicate that TrainerWall’s integrated approach effectively addresses three critical healthcare challenges: rapid deployment of isolation facilities, standardization of infection control procedures, and enhancement of healthcare worker training. The demonstrated efficacy of its antimicrobial materials and automated sanitization in maintaining sterile environments, along with its adaptable modular design, has significant implications for improving global health infrastructure—especially in regions where permanent isolation facilities are unfeasible.

By aligning with best practices in infection prevention and control, TrainerWall holds promise for long-term improvements in healthcare delivery, particularly in low-resource settings. Its portability and adaptability offer a valuable tool for enhancing global healthcare infrastructure, especially in regions most vulnerable to infectious disease outbreaks.

Future research priorities include conducting longitudinal studies to quantify infection prevention rates across diverse healthcare settings, evaluating cost-effectiveness through comparative analyses with traditional isolation methods, assessing user satisfaction and training effectiveness using standardized metrics, and measuring the impact of augmented reality integration on protocol compliance. For practical implementation, healthcare facilities are advised to establish comprehensive deployment protocols, develop standardized training programs, implement regular maintenance schedules, and create monitoring systems to track usage patterns and outcomes. In addition, the development of region-specific guidelines that consider local resource availability and healthcare needs is recommended.

In summary, TrainerWall has the potential to become a standard component of infection prevention strategies worldwide, significantly contributing significantly to global efforts to control infectious diseases while enhancing healthcare quality and accessibility in the areas most in need.

## 7. Patents

Belardi, M.; Cennamo, V.; Cornacchia, G.; Ramorino, G. Removable Antechamber for the Isolation of Pathogens in Health Facilities (Anticamera Smontabile per l’isolamento Di Patogeni in Ambienti Di Strutture Sanitarie) N. 102020000006505, 2020.

## Data Availability

The original contributions presented in this study are included in the article. Further inquiries can be directed to the corresponding author(s).

## Appendix A

The recommended protocol for healthcare personnel when using Personal Protective Equipment (PPE) is designed to ensure thorough protection and minimize the risk of contamination. This procedure involves several critical steps:

Donning Procedure:

1. Preparation: Prior to donning PPE, healthcare workers should remove all personal items, including jewellery, and perform hand hygiene with soap and water or an alcohol-based sanitizer.
2. Footwear: Protective shoe covers are donned to prevent contamination from surfaces.
3. PPE Integrity Check: Inspect all PPE components (gloves, gown, mask, goggles) for any defects. Damaged items should be discarded and replaced.
4. Initial Glove Application: The first pair of gloves is worn, ensuring proper fit and coverage.
5. Gown and Respiratory Protection: A disposable gown is worn over the uniform, followed by facial filtering protection (FFP2/FFP3 mask). Protective goggles are then donned.
6. Secondary Glove Application: A second pair of gloves is applied over the first pair, providing an additional layer of protection.

Doffing Procedure:

7. Minimize Contact: During doffing, avoid touching the face, mucous membranes, or skin with potentially contaminated PPE.
8. Disposal: Place disposable PPE (gowns and gloves) in designated biohazard containers. Reusable items, such as goggles, should be sanitized promptly.
9. Sequence of Removal:
  - Remove and discard the gown, rolling it inward to contain contamination.
  - Remove and dispose of the first pair of gloves.
  - Carefully remove and sanitize goggles.
  - Remove the FFP2/FFP3 mask by handling it from the back and dispose of it.
  - Remove shoe covers and the second pair of gloves, followed by hand hygiene with soap and water or an alcohol-based sanitizer.
